# Hepatic Resection as a Safe and Effective Treatment for Hepatocellular Carcinoma Involving a Single Large Tumor, Multiple Tumors, or Macrovascular Invasion: Erratum

**DOI:** 10.1097/01.md.0000464211.01415.50

**Published:** 2015-02-13

In the article “Hepatic Resection as a Safe and Effective Treatment for Hepatocellular Carcinoma Involving a Single Large Tumor, Multiple Tumors, or Macrovascular Invasion,^1^ which appeared in Volume 94, Issue 3 of *Medicine*, the word “disorder” was incorrectly substituted for the word “pathology” in the first sentence of the Definitions section. Also, in Figure 3, the y-axis incorrectly read “Overall survival (%)”. It should have read “Disease-free survival (%)”. The article has since been corrected online.
